# Case Report: Clinicopathological features of papillary renal neoplasm with reverse polarity: a report of four patients and literature review

**DOI:** 10.3389/fonc.2026.1903759

**Published:** 2026-07-29

**Authors:** Raymond Yu O, Anthony Wing Ip Lo, Hoi Lung Wong

**Affiliations:** 1 Department of Pathology, Li Ka Shing Faculty of Medicine, The University of Hong Kong, Hong Kong, Hong Kong SAR, China; 2 Division of Anatomical Pathology, Department of Pathology, Queen Mary Hospital, Hong Kong, Hong Kong SAR, China; 3 Division of Urology, Department of Surgery, Queen Mary Hospital, Hong Kong, Hong Kong SAR, China

**Keywords:** diabetes mellitus, hyperglycaemia, oncology, papillary renal neoplasm with reverse polarity (PRNRP), renal cancer, renal cell carcinoma, renal neoplasm

## Abstract

Papillary renal neoplasm with reverse polarity (PRNRP) is an emerging entity with an indolent clinical course. It is histologically characterized by a low-grade papillary neoplasm with apically located nuclei and frequent *KRAS* mutation. With the limited amount of literature, there is no established association between PRNRP and other diseases. Herein, we report four patients of PRNRP in Hong Kong. The neoplasms are discovered incidentally by radiographic investigations and diagnosed by their classical histopathological features. All patients are noted to have underlying hyperglycaemia en passant. This preliminary observation provides insight into the pathogenesis of this specific type of renal neoplasm.

## Introduction

Papillary renal neoplasm with reverse polarity (PRNRP) is an emerging entity with more than 300 cases reported. Typically, the tumour has an indolent clinical course and no metastasis or disease-specific death has been reported (1). It is usually discovered incidentally in imaging studies and diagnosed by histopathologists after excision. The tumour shows distinct histological features, displaying low-grade apically located nuclei within the oncocytic tumour cells arranged in papillary architecture (1–3). Typically, the tumour cells show strong GATA3 immunohistochemical expression. *KRAS* mutation is the most common genetic mutation in PRNRP, occurring in more than 80% of cases (1).

To date, more than 300 cases have been reported, characterizing its histopathological, immunohistochemical and molecular features. Nevertheless, the pathogenesis of the tumour remains largely uncertain. A recent study showed that PRNRP expressed genes in the mTOR signaling pathway and that biological processes in oxidative phosphorylation and fatty acid metabolism were enriched in PRNRP (4). However, the clinical correlation of the mTOR signaling pathway in the context of PRNRP has yet to be established.

Diabetes mellitus (DM) can be diagnosed using various criteria, including a fasting glucose level of 7.0 mmol/L or higher (5). A fasting glucose level between 5.6 and 7.0 mmol/L can be classified as pre-DM (5), and a level above 5.6 mmol/L can be considered as hyperglycaemia. It is known that the mTOR signaling pathway is associated with DM (6). In this regard, it is tempting to speculate that hyperglycaemia can lead to activation of the mTOR pathway, subsequently leading to the pathogenesis of PRNRP.

Here we report four cases of PRNRP in Hong Kong. Among the four patients, three have DM, and the remaining patient has pre-DM. Possible links between this emerging entity and hyperglycaemia are discussed to provide insight into the pathogenesis of this specific type of renal cell carcinoma.

## Materials and methods

### Patients and sample

All patients who underwent partial or radical nephrectomy and received a diagnosis of PRNRP or papillary renal cell carcinoma from January 2015 to March 2026 were identified in the internal database for routine reporting of histopathology services by the Division of Anatomical Pathology at Queen Mary Hospital, Hong Kong. All specimens with formalin-fixed, paraffin-embedded (FFPE) tissue blocks were included. Haematoxylin and eosin (H&E)-stained slides and immunohistochemically stained slides were reviewed by two pathologists (R.Y.O and A.W.I.L). Clinical information on each patient from the electronic medical records, the operation records and the pathological reports, including the sex, age, tumour characteristics, TNM stage, fasting glucose level, medication records, latest follow-up status and clinical progression were retrieved and anonymized.

### Histopathological analysis

Pathological reports, microscopic, immunohistochemical features and molecular analyses of the selected cases were reviewed in detail, including tumour architecture, nuclear grading, cytoplasm, immunohistochemical stains, surrounding parenchyma and other pathological findings. Targeted sequencing of KRAS exon 2 of the tumour DNA was performed in the routine service molecular pathology lab of our division in Queen Mary Hospital. This lab is accredited by the College of American Pathologists, in which KRAS hot spot analysis by PCR and Sanger sequencing was listed in the service menu.

## Results

### Cases

**Figure 1:**
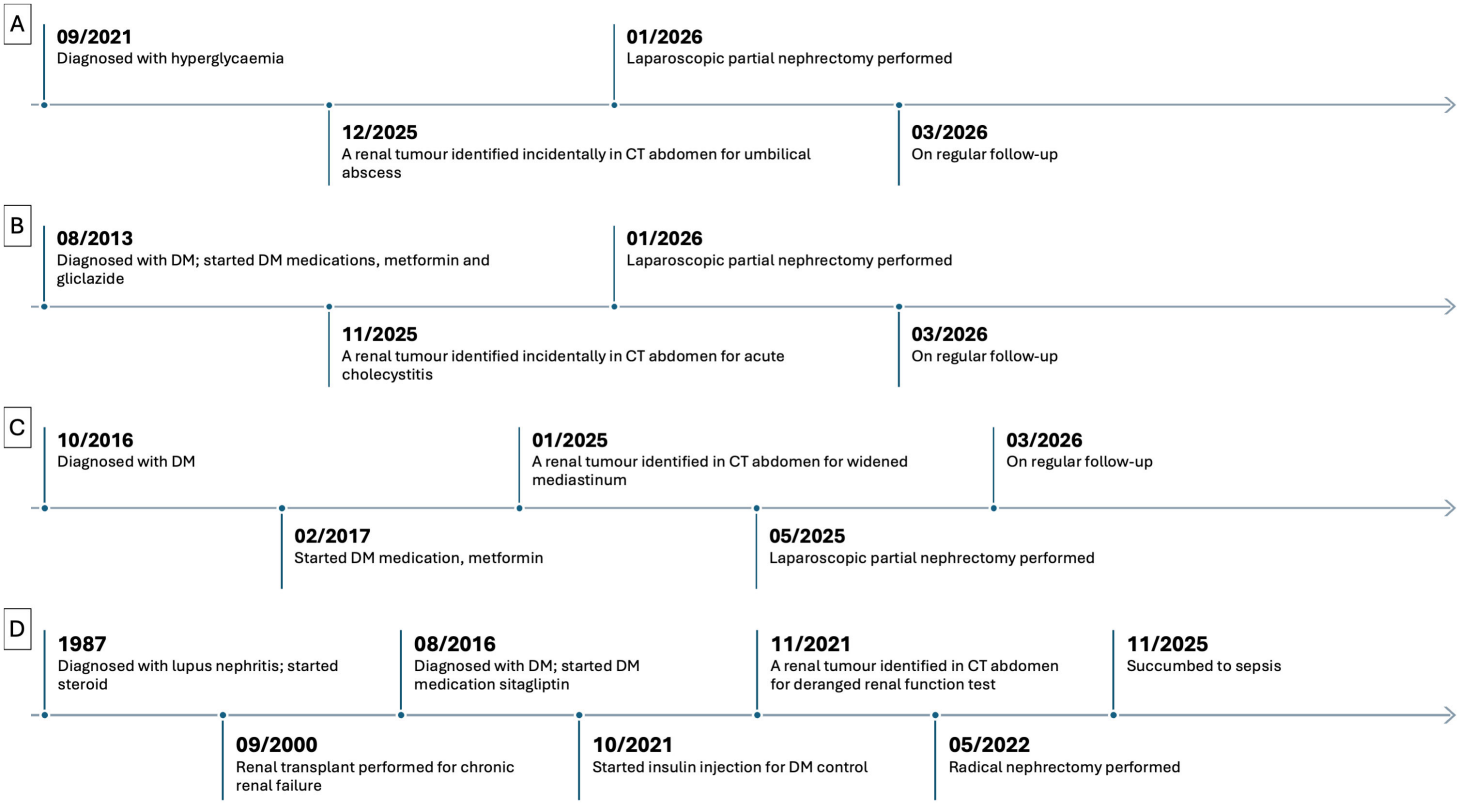
Timelines including pathways from the diagnosis and treatment of hyperglycaemia to the detection and treatment of PRNRP of **(A)**, Patient 1; **(B)**, Patient 2; **(C)**, Patient 3 and **(D)**, Patient 4.

The clinicopathological features of the four cases are summarized in **Table 1** and **Figure 1**.

**Table 1:** Clinicopathologic features of 4 patients with PRNRP.

	Case 1	Case 2	Case 3	Case 4
Sex	F	M	F	F
Age (year)	62	53	81	54
Tumour-related symptoms/signs	Nil	Nil	Nil	Nil
Operation performed	Laparoscopic partial nephrectomy	Laparoscopic partial nephrectomy	Laparoscopic partial nephrectomy	Radical nephrectomy
Tumour characteristics				
Size (cm)	1.2	1.6	1.6	2.0
Laterality	Left	Left	Left	Right
Gross appearance	Solid-cystic	Solid-cystic	Cystic	Nodular
Architectural feature	Papillary	Papillary	Papillary	Papillary
Cytological feature	Oncocytic cells with apically located nuclei	Oncocytic cells with apically located nuclei	Oncocytic cells with apically located nuclei	Oncocytic cells with apically located nuclei
WHO/IUSP histologic grade	1	1	1	1
GATA3 IHC	+	+	+	+
KRAS mutation	G12V	Negative	G12V	G12V
Pathologic T-stage	1a	1a	1a	1a
Patient clinical conditions				
Obesity/overweight	+	+	+	–
BMI	36	25.4	27.5	16.6
Hypertension	+	+	+	–
Hyperlipidaemia	–	+	+	–
Hyperglycaemia	+	+	+	+
Duration of hyperglycaemia until tumour detection by CT (months)	52	148	100	64
Most recent fasting glucose (mmol/L)	5.9	6.6	5.7	4.3
Most recent haemoglobin A1c level (reference range: 4.0-6.0%)	Nil	8.0%	6.1%	5.4%
DM medications	Nil	Metformin and gliclazide	Metformin	Insulin injection
Other medical conditions	Obstructive sleep apnea	Fatty liver; psoriasis vulgaris	Nil	Lupus nephritis; post-renal transplant on immunosuppressives
Follow up (months)	2	2	10	42
Update	Alive	Alive	Alive	Death/other causes

+, indicates positive; -, negative; F, female; M, male.

### Patient information and clinical findings

Of the four patients, three were female, and one was male. At the time of diagnosis, their age ranged from 53 years to 81 years (**Table 1**). Clinically, they suffered from various metabolic diseases, including overweight or obesity, hypertension and hyperlipidaemia. Notably, all patients had hyperglycaemia, and three of them were on diabetic medications, including insulin injection. Patient 4 had a low body mass index and no other metabolic diseases apart from diabetes mellitus. She was a 54-year-old female with a long history of lupus nephritis and end-stage renal disease. She received a renal transplant at 33 years old and subsequently was on long-term immunosuppressive medications. She was diagnosed with DM at 48 years old because of the side effects of the immunosuppressive medications.

**Figure 2:**
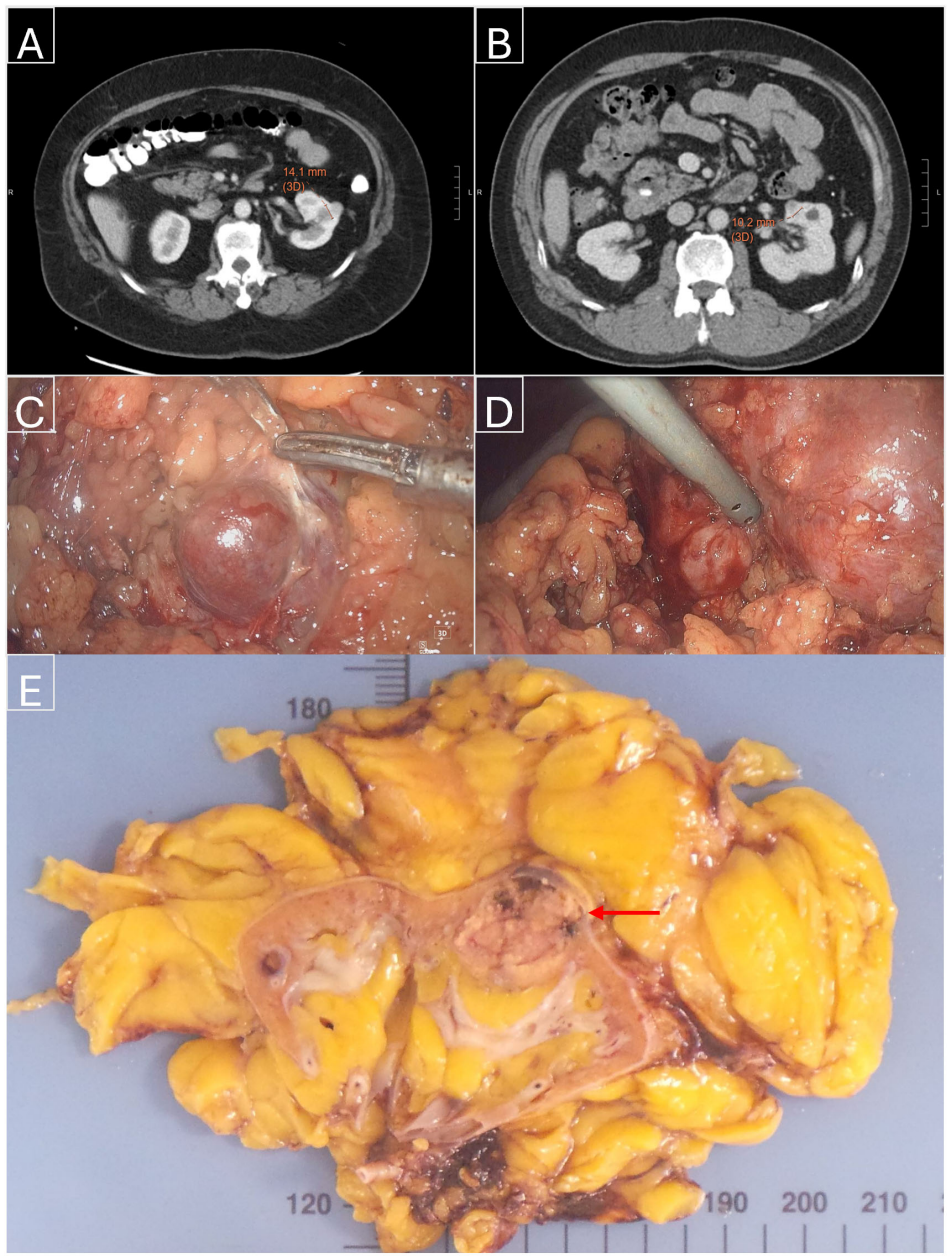
Contrast-enhanced CT, intraoperative and gross images of selected patients. **(A, B)** Contrast-enhanced CT revealed a 1.4 cm tumour in the middle pole of the left kidney in patient 1 and a 1.0 cm lesion in the anterior middle pole of the left kidney in patient 2, respectively. **(C, D)** Intraoperatively, a well-circumscribed cystic tumour was found at the lateral side of the left kidney in patient 1 and near the hilar region of the left kidney in patient 2, respectively. **(E)** A nodular tumour (arrow) was identified in the middle pole of the right native kidney of patient 4, who was diagnosed with lupus nephritis and end-stage kidney disease.

None of the patients complained of urological or mass symptoms or signs. Physical examinations were all unremarkable. The tumours were all detected incidentally by imaging (**Figures 2A, B**) for other purposes, including umbilical abscess, acute cholecystitis, widened mediastinum and deranged renal function test upon follow-up of renal transplant.

On imaging, the tumours ranged from 1.2 to 2.0 cm in maximal dimension. Three of them were situated in the left kidney and one in the right kidney. No evidence of metastasis was seen in all cases.

### Therapeutic intervention

Laparoscopic partial nephrectomy was performed in three cases, and case 4 had undergone radical nephrectomy because of end-stage renal disease (**Figures 2C, D**). The surgical operations were all uneventful. No adjuvant therapy was given to all patients.

### Histopathological and genetic study

**Figure 3:**
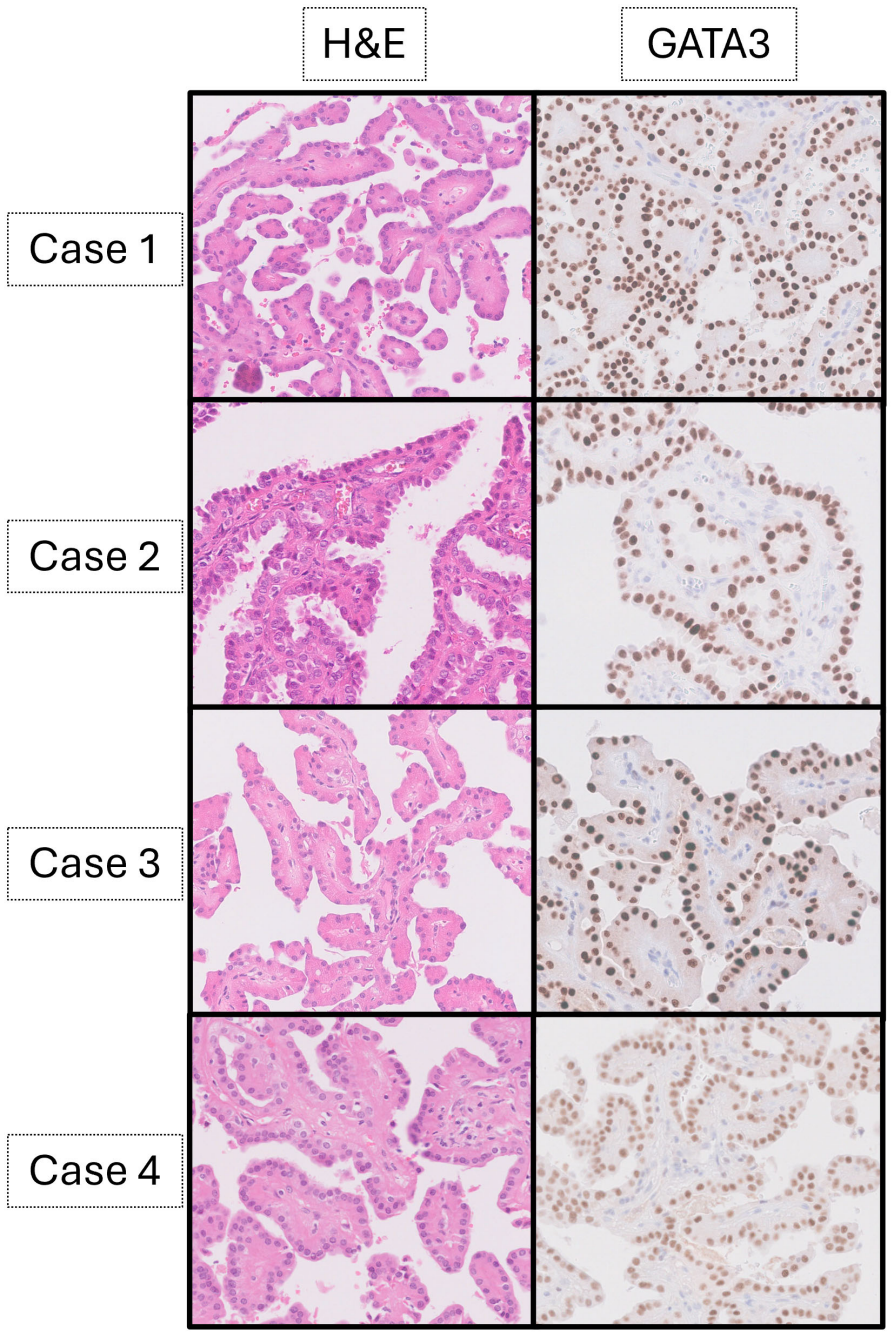
Histopathological (magnification = 220x) and GATA3 immunohistochemical features (magnification = 220x) of the four cases.

Upon gross examination, the tumours had either cystic, solid-cystic or nodular appearance (**Figure 2E**). Histologically, all tumours are composed of oncocytic cells arranged in papillary architecture with apically located nuclei (**Figure 3**). The tumour cells have a low-grade nuclear feature and show strong GATA3 positivity. They all have a T1a pathologic stage. Notably, the *KRAS* G12V mutation was also detected in three cases (**Table 1**).

### Outcome

The patients had a follow-up period ranging from 2 to 42 months. All patients had no significant surgical complications or tumour recurrence. Three of them were on regular surveillance. Patient 4 has succumbed to sepsis owing to immunosuppressive therapy for underlying lupus nephritis and renal transplant, 42 months post-operation.

### Literature review

Most previous case reports and studies do not mention the patients’ past medical history (**Table 2**). Among twenty-five patients (including the four patients in this study) whose medical history could be traced, eight have been specifically reported to suffer from underlying DM (32%). However, blood glucose levels are unknown in all reports and studies, precluding the identification of pre-DM patients.

**Table 2:** Cases with reported DM in the literature.

Year	Author	Number of cases reported	Number of cases with DM
2026	Our study	4	3
2026	Lee et al. (1)	77	Not mentioned
2025	Liu et al. (20)	1	Not mentioned
2025	Eryilmaz et al. (21)	1	Not mentioned
2025	Sanmiguel et al. (4)	17	Not mentioned
2025	Chen et al. (22)	1	0
2025	Gonzalez et al. (18)	1	1
2025	Nithagon et al. (23)	1	0
2025	Vangapandu et al. (24)	1	Not mentioned
2025	Kannan et al. (17)	1	1
2025	Xing et al. (25)	1	0
2025	Wang et al. (26)	35	Not mentioned
2025	Gonda et al. (27)	2	Not mentioned
2025	Lobo et al. (28)	1	Not mentioned
2025	Wen et al. (29)	26	Not mentioned
2024	Shao et al. (30)	2	Not mentioned
2024	Nemours et al. (12)	10	Not mentioned
2024	Han et al. (31)	9	Not mentioned
2024	Castillo et al. (32)	8	Not mentioned
2023	Tu et al. (33)	1	0
2023	Nova-Camacho et al. (34)	8	Not mentioned
2023	Kim et al. (8)	43	Not mentioned
2022	Yuzhi et al. (35)	2	Not mentioned
2022	Yang et al. (36)	11	Not mentioned
2022	Wei et al. (37)	7	Not mentioned
2022	Wang et al. (19)	15	3
2022	Shen et al. (38)	16	Not mentioned
2022	Liu et al. (39)	20	Not mentioned
2022	Al-Obaidy et al. (40)	50	Not mentioned
2021	Pivovarcikova et al. (41)	2	Not mentioned
2021	Kiyozawa et al. (42)	14	Not mentioned
2021	Chang et al. (43)	10	Not mentioned
2020	Tong et al. (44)	10	Not mentioned
2020	Lee et al. (45)	1	Not mentioned
2020	Kim et al. (46)	30	Not mentioned

## Discussion

Here, we report four cases of PRNRP in Hong Kong. These cases have the typical morphological, immunohistochemical and clinical features of PRNRP, which is a low-grade papillary oncocytic tumour with GATA3 expression and frequent *KRAS* mutation and with an excellent prognosis.

The diagnosis of PRNRP relies mainly on histopathological examination with ancillary immunohistochemical staining. According to the WHO classification of tumour, PRNRP is currently considered to be a variant of papillary renal cell carcinoma (7). Hence, papillary renal cell carcinoma is the major differential diagnosis of PRNRP. The key differences between the two entities lie on the apically located tumour cell nuclei, the presence of GATA3 expression and the absence of CAIX expression in PRNRP. These key morphological and immunohistochemical features were present in our four cases (**Figure 3**), confirming the diagnosis of PRNRP.

There is no established linkage between PRNRP and other diseases, and the pathogenesis of PRNRP is still uncertain, despite the identification of the typical *KRAS* mutation. An earlier study has discovered the possible involvement of MAPK/ERK and RALGDS pathways, by showing high p-ERK, RalA, and RalB protein expression via immunohistochemical staining in PRNRP (8).

A recent study has demonstrated that PRNRP would express genes of the mTOR signaling pathway (4), which is known to be closely related to DM (6). High glucose level in DM patients could lead to the activation of mTORC1, which in turn, promotes anabolic growth of cells, whereas the high insulin level in early DM patients could initiate the mTORC2 pathway, resulting in cell proliferation and survival (6). Importantly, alterations of mTOR genes are also identified in other low-grade, newly-emerging oncocytic renal cell neoplasms such as eosinophilic solid and cystic renal cell carcinoma (9), low-grade oncocytic tumour (10) and eosinophilic vacuolated tumour (11).

In another study, PRNRP has shown a differential expression of various non-coding RNAs. There is an over-expression of *miR-155*, which is closely related to the hypoxic signaling pathway (12). Intriguingly, *miR-155* has been identified as a key player in the pathogenesis of DM and its therapeutic potential in DM is under extensive investigation (13). The *miR-155* expression levels appear to vary in different organs and are crucial to the end-organ damage caused by DM. In the kidney, over-expression of *miR-155* has been demonstrated to trigger diabetic nephropathy via several signaling pathways, one of which is the mTOR pathway (13).

It appears that both DM and hypoxic signaling pathways are implicated in the tumorigenesis of various tumours, including renal malignancies. A large cohort study has demonstrated that women with DM had a significantly increased risk of renal cell carcinoma, compared with those without DM (14). Meanwhile, the prescription of the anti-diabetic medication sodium glucose cotransporter-2 inhibitors (SGLT2-inhibitors) could reduce the risk of renal cell carcinoma (15). It has also been studied that the advanced glycation end products secondary to hyperglycaemia are crucial for the oncogenic *KRAS*-mediated hypoxic signaling in pancreatic cancer (16). Considering that *KRAS* mutation is common in PRNRP, these findings cast light on the possible association of hyperglycaemia and PRNRP.

Notably, the four patients we reported all suffer from hyperglycaemia with various metabolic diseases. Three recent case reports and studies also described cases of PRNRP patients with underlying DM (17–19). Yet, most other literatures have not specifically reported DM or hyperglycaemia in their PRNRP cases, rendering the prevalence of hyperglycaemia among PRNRP patients unknown. Our cases serve as a preliminary observation of the linkage between hyperglycaemia and PRNRP. Further studies and review of previous case series will help in confirming or rebutting this association. Studying this rare entity would enlighten some of the specific pathways leading to the development of renal malignancies.

However, with only four cases in Hong Kong, the observation may not be generalizable to the broader population. Also, this case report does not include a comparison group, and the observed association does not imply causation. Further studies are needed to explore any potential linkage between hyperglycaemia and PRNRP.

## Data Availability

The original contributions presented in the study are included in the article/supplementary material, further inquiries can be directed to the corresponding author/s.
